# A case of dermatophyte abscess and adjunctive use of a novel RNA: In situ hybridization to confirm *Trichophyton rubrum*

**DOI:** 10.1016/j.jdcr.2024.01.027

**Published:** 2024-02-07

**Authors:** Andrea Marie Bernales-Mendoza, Akira Shimizu, Takashi Mochizuki, Kazushi Anzawa, Reimon Yamaguchi, Kiminobu Takeda

**Affiliations:** a Dermatology Department, Kanazawa Medical University, Ishikawa Prefecture, Japan; b Jose R. Reyes Memorial Medical Center, National Specialty Center for Dermatology, Manila, Philippines; c Dermatology Department, Jose N. Rodriguez Memorial Hospital and Sanitarium, Caloocan, Philippines

**Keywords:** dermatopathology, dermatophyte, fungus, infectious diseases, molecular biology, mycology

## Introduction

*Trichophyton rubrum* has been known to elicit a range of clinical infections from superficial to deep mycosis. Histologic identification of fungal elements remains difficult.^1^ Special stains like Grocott-methenamine silver and periodic acid–Schiff increase the detection^2^ but the gold standard remains culture and morphologic identification of the fungi itself.^1^ In situ hybridization (ISH) is a method being used for fungal identification and as such, refinements on the technique have been developing.^3^, ^4^, ^5^, ^6^^,^^7^ We present a case of dermatophyte abscess caused by *T rubrum* detected in formalin-fixed paraffin-embedded tissue using conventional fungal stains and compared with a novel RNA—ISH technique.

## Case report

**Fig 1:**
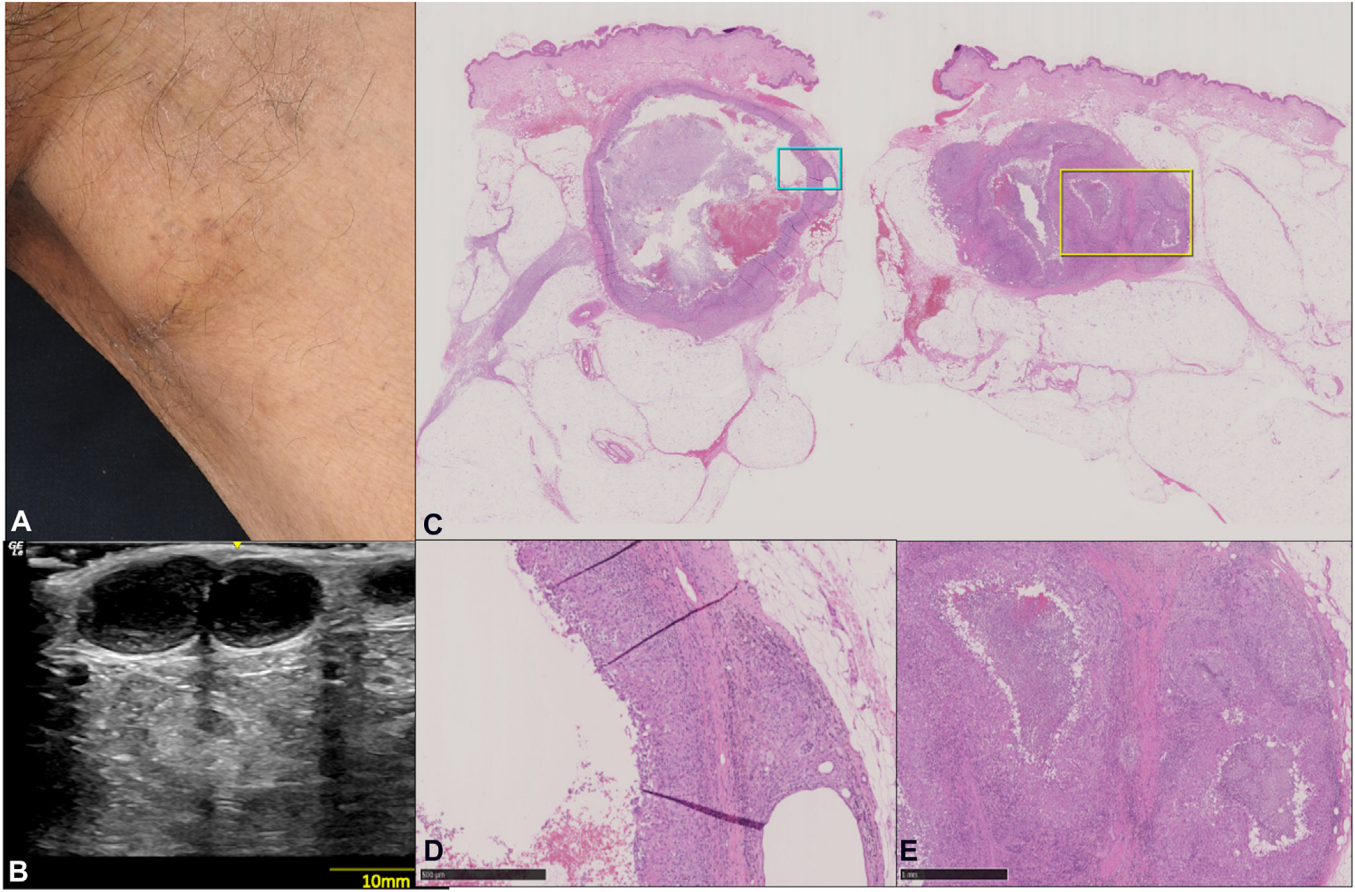
**A,** Multiple discrete, slightly mobile, firm, nontender subcutaneous nodules measuring 3 cm in largest diameter with overlying skin noted with fine scaling at the left inguinal region. **B,** Ultrasound revealed the multilocular cavities within the dermis extending up to the subcutaneous layer. **C,** H and E tissue sections showing a pseudocystic lesion in the dermis extending to the subcutis. **D,** 40× magnification of *blue box* in C: Formation of a fibrous capsule with multinucleated giant cells. **E,** 40× magnification of *yellow box* in C: Granulomatous reaction surrounding the cavity.

A 63-year-old man presented with multiple nodules in the left inguinal area of 3 months duration. He had a history of rheumatoid arthritis on prednisolone 6 mg/d and tacrolimus 1 mg/d. He had been applying topical steroid (difluprednate) ointment to the groin for pruritus for 5 months. On physical exam, lesions were observed to be multiple slightly mobile, firm nontender nodules measuring 3 cm in largest diameter with fine scaling on overlying skin at the left inguinal region. No punctum, erythema, or gross purulent discharge on other areas of the body affected. Ultrasound of the inguinal lesion revealed multilocular cavities within the dermis extending up to the subcutis (Fig 1).

**Fig 2:**
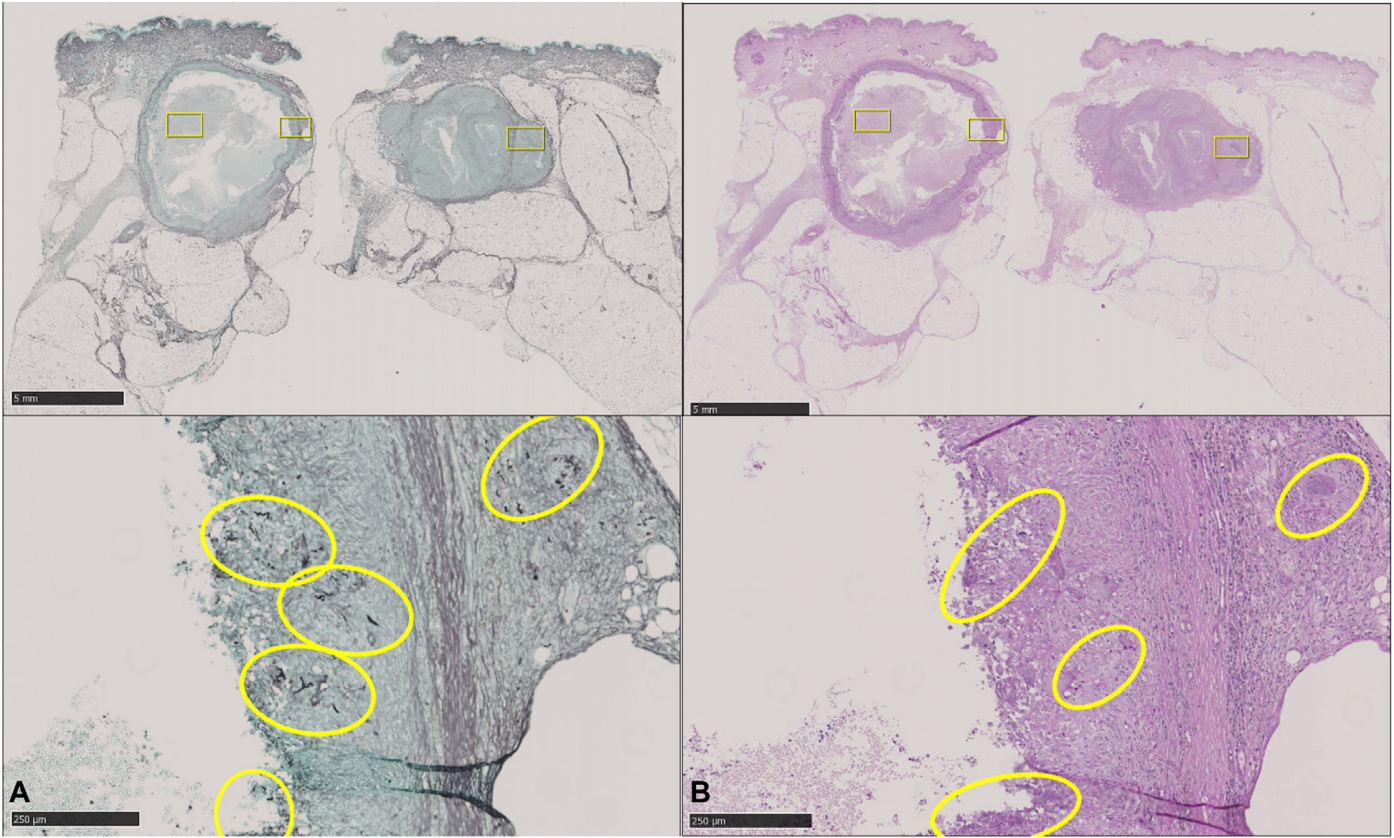
**A,** Grocott-methenamine silver stain. **B,** Periodic acid–Schiff stain; *boxes*/*circles* show the hyphal elements.

**Fig 3:**
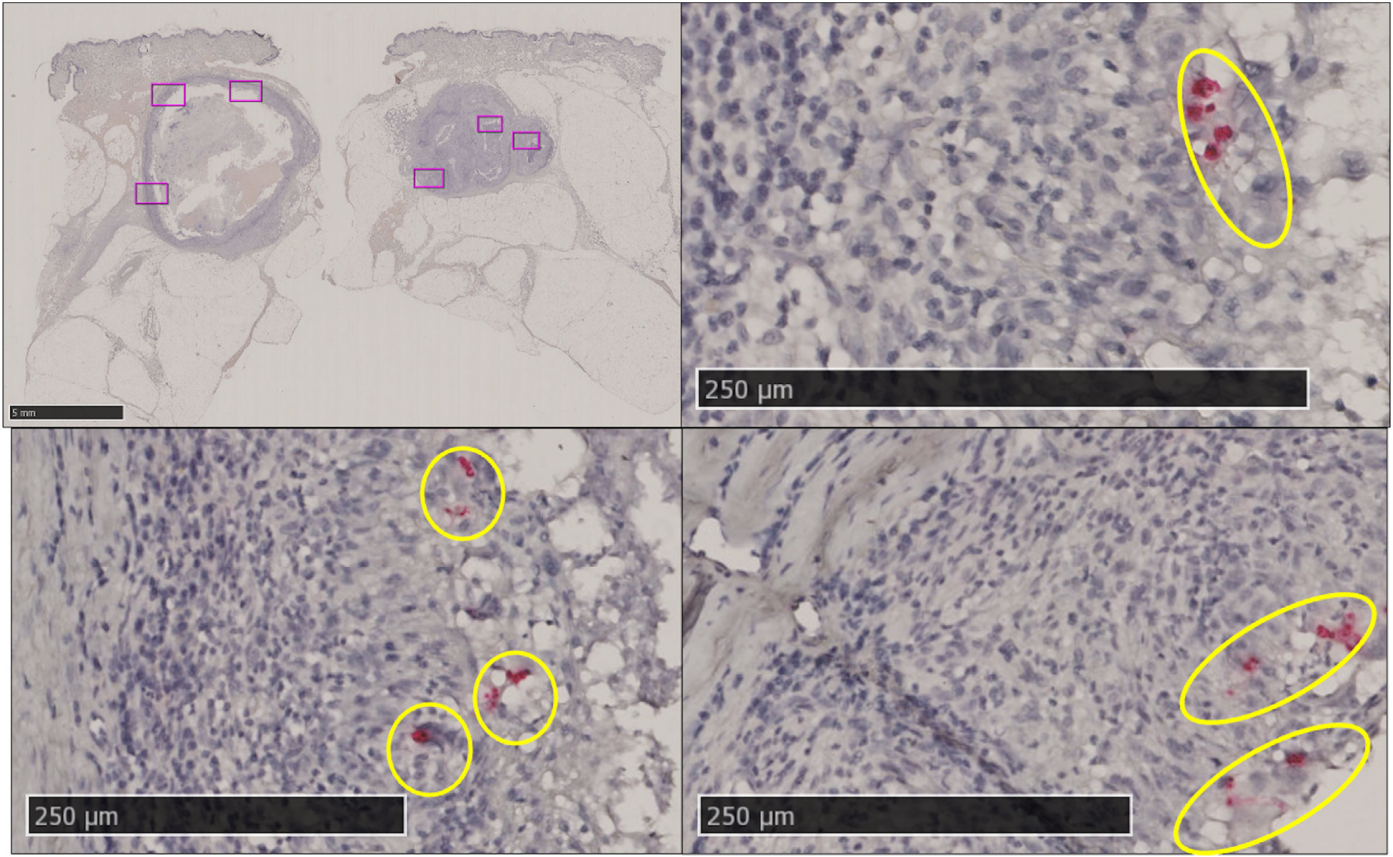
RNAscope-ISH stain: Positive stain indicated as *boxes*. Red punctate signals on closer magnification (highlighted in *circles*).

Direct microscopic examination of the pus extracted from the nodules revealed fungal elements and upon further confirmatory tests including KOH, fungal culture, and internal transcribed spacer —gene sequencing confirmed *T rubrum*. The same fungus was isolated from scale of overlying skin using the same methods described above. A biopsy revealed a pseudocystic cavity surrounded by a fibrous capsule with granulomatous reaction containing numerous multinucleated giant cells. Periodic acid–Schiff and Grocott-methenamine silver demonstrated hyphal elements within and around the wall (Fig 2). RNA-ISH detected fungal gene expression within and around the wall as well (Fig 3). Oral terbinafine was given at 125 mg/d for 6 weeks. The nodules completely resolved by the sixth week of treatment with β-D glucan levels returning to baseline (<11 pg/mol) from a level of 21.2 pg/ml at initial workup. Repeat ultrasound on follow up showed no residual infection or recurrence at 18 months.

## Discussion

The case described above demonstrated how a patient developed invasive dermatophytosis while on oral immunosuppressive medications and concurrent topical steroid application.

A systematic review concluded that immunosuppression, together with misuse of topical steroids, were seen as factors causing further invasion of an infection into a hair follicle.^8^ Additionally, *T rubrum* was recognized as the most common cause of invasive dermatophytosis.^8^

“Dermatophyte abscess” first used by Fukushiro^9^ was used to describe a pseudocystic mass deep in the dermis caused by dermatophytes. Dermatophyte abscess, classified as a type of deeper dermatophytosis, is distinct from Majocchi granuloma (fungal folliculitis) as it affects deeper layers of the skin. It has extensive inflammation of the dermis and subcutis with destruction of the hair follicle and collagen degradation. Because this entity is mainly seen in immunocompromised patients, it has the potential to cause life-threatening and/or disseminated infections.^8^

Microbiologic and molecular methods are now used in conjunction to confirm the diagnosis of fungal infection.^10^ Molecular-based techniques like polymerase chain reaction and ISH give the highest accuracy in this regard.

This study, to our knowledge, is the first to employ the new ISH technique to identify dermatophytes, specifically, *T rubrum.* RNAscope-ISH (ACD Bio) is a commercially available ISH kit which uses the drop-bottle method. Together with a shorter processing time, it is able to detect signals from very low expressing genes as well as partially degraded material. The enhanced sensitivity is due to the double Z probe that enables expression of signal from single-molecule RNA genes of any species. It has become widely used in recent years for a wide range of applications and is beginning to be used to aid in clinical diagnosis through automation.^7^ Accessibility to this technology may become easier due to its innately simplified equipment use, steps, and interpretation.

This method has low risk of contamination compared to polymerase chain reaction, minimal background noise compared to special stains, and faster accurate result compared to fungal culture. The structural and morphologic context it provides also has an advantage for better clinicopathologic correlation.

The probe designed for this study was from the large subunit of the ribosomal RNA of *T rubrum* (Accession number: JX431933). Targeted probe region: CGGGCGCGTTGGGCCCCGGGCGGAGGCCCCCGAGGCAAGGCGGCACTAGCCGGGAGACCGG.

**Fig 4:**
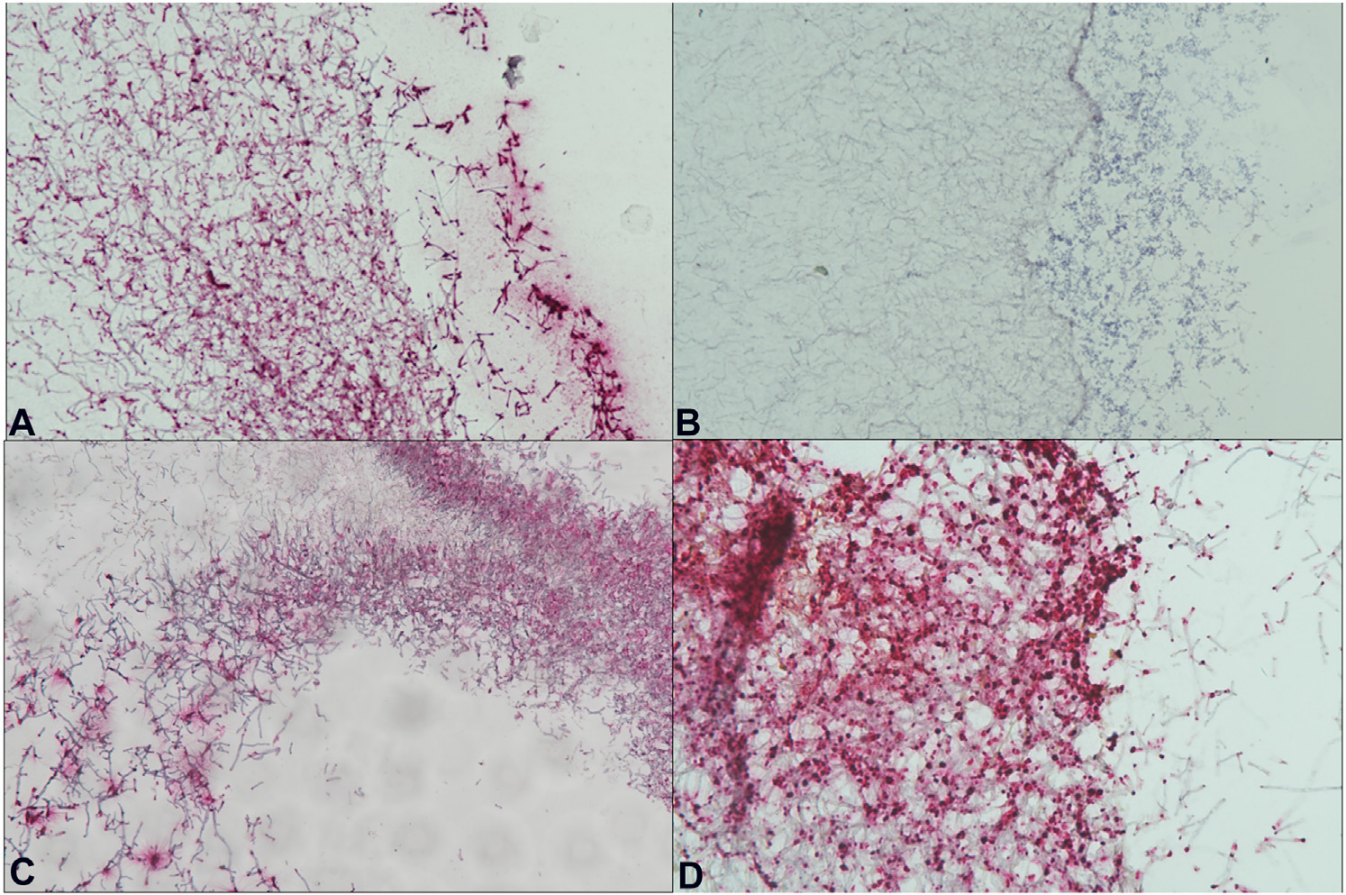
Agar blocks tested with RNAscope-ISH stain: **A,***Trichophyton rubrum*. **B,***Trichophyton interdigitale*. **C,***Trichophyton violaceum*. **D,***Trichophyton soudanense*.

Preliminary determination of the probe’s specificity used agar blocks (Fig 4). It distinguished *T rubrum* from *Trichophyton interdigitale*, a diagnosis that previously can only be confirmed by a combination of culture techniques and polymerase chain reaction. This is important since the former is known to be the most prevalent pathogen causing invasive dermatophytosis^8^ Phylogenetic studies on the *T rubrum* complex is continuously evolving and therefore its ability to correctly identify it as well as other genetically homologous species within the same group, *Trichophyton violaceum* and *Trichophyton soudanense* is invaluable. This ISH method may have reached its highest possible specificity for research and clinical use.

To validate the results, the probe sequence was compared with the sequences of the tested *Trichophyton* species. It was determined that there was only one (1) base pair difference between the probe sequence of *T rubrum* and the probe region sequence of the 2 *T rubrum* complex species, *T violaceum* and *T soudanense*. Additionally, there had been six (6) base pair differences in the probe sequence of *T rubrum* vs the probe region sequence of *T interdigitale*.

This ISH method is intended only as adjunct for dermatopathology to confirm a specific fungal infection. Each probe should be used together with a complete knowledge of the clinical case and related laboratory results.

In conclusion, dermatophyte abscess is a rare presentation of deep dermal dermatophytosis. Predisposing factors need to be identified on patients who are at risk to develop such a condition. RNAscope - ISH technique has the advantage of faster, accurate species-identification of cutaneous fungal infection.

## Conflicts of interest

None disclosed.
